# Efficacy and safety of respiratory strength and endurance training in patients with myotonic dystrophy type 1 (DM1): a randomized controlled trial

**DOI:** 10.1007/s00415-025-13362-z

**Published:** 2025-09-11

**Authors:** Stephan Wenninger, Eva Heidsieck, Corinna Wirner-Piotrowski, Marko Mijic, Natalia Garcia-Angarita, Kristina Gutschmidt, Daniel H. Mendelshohn, Benedikt Schoser

**Affiliations:** Department of Neurology, Friedrich-Baur-Institute, Ludwig-Maximilians University Munich, Ziemssenstr. 1, 80336 Munich, Germany

**Keywords:** DM1: Myotonic Dystrophy Type 1, IMT: Inspiratory mucle training, Respiratory muscle training, Endurance training

## Abstract

**Background:**

Myotonic dystrophy type 1 (DM1) is a multisystem disorder characterized by progressive muscle weakness, including the respiratory muscles, which often leads to ventilatory insufficiency. Despite its clinical relevance, high-quality controlled data on the effects of respiratory muscle training in DM1 are limited.

**Objective:**

To evaluate the safety and effectiveness of long-term, home-based inspiratory muscle training (IMT) using a commercially available inspiratory training device in genetically confirmed DM1 patients.

**Methods:**

This was a single-center, randomized controlled trial involving three parallel groups: inspiratory strength training, inspiratory endurance training, and a control group without training. Patients were followed over 9 months. The primary outcome was the change in maximal inspiratory pressure (MIP) after nine months. Secondary outcomes included forced vital capacity, maximal expiratory pressure, capillary blood gases, a 6-min walk test, and validated patient-reported outcomes. Adherence and safety were monitored.

**Results:**

Twenty-six participants completed the study. The intervention was safe, with no serious adverse events. Significant improvements in primary outcome MIP were observed in both intervention groups compared to control (*p* < 0.05), with the strength group showing the greatest benefit. Secondary outcomes improved significantly in the endurance group. Adherence to the training exceeded 80% across all groups. Baseline differences in MIP, FVC, and FEV1 were observed and considered in the analysis.

**Conclusion:**

Regular IMT is a feasible, safe, and effective intervention for improving respiratory function in patients with DM1. Regular RMT can enhance respiratory muscle strength and endurance and should be incorporated into the multidisciplinary care of DM1 patients showing initial signs of restrictive ventilatory insufficiency.

**Supplementary Information:**

The online version contains supplementary material available at 10.1007/s00415-025-13362-z.

## Introduction

Myotonic dystrophy type 1 (DM1) is a chronic progressive, autosomal dominant, inherited multisystem disorder caused by a mutation in the DMPK gene, leading to variable CTG-repeat expansions [1]. It is the most prevalent muscular dystrophy in adults, with a prevalence of around 10/10,000 [2]. Clinical hallmarks are myotonia, progressive muscle weakness with predominant distal distribution, and a variety of systemic manifestations, including cardiac, endocrine, gastrointestinal, and cognitive impairments. Clinical onset and disease course are roughly associated with CTG repeat expansion [3–6]. One of the most debilitating and life-limiting aspects of DM1 is the progressive involvement of respiratory muscles, resulting in alveolar hypoventilation, ineffective cough, and increased susceptibility to respiratory infections. Natural history studies revealed an estimated annual decline in forced vital capacity (FVC) of 0.73% [7]. Clinically, patients report severe reductions in quality of life due to fatigue, hypersomnolence, and reduced exercise tolerance [4, 8]. Moreover, respiratory involvement significantly contributes to morbidity and premature mortality. In this multisystemic disorder, patients may also develop central or obstructive sleep apnea, which might add to restrictive ventilatory insufficiency [8–10]. Current therapeutic strategies are essentially symptomatic and focused on managing complications. Respiratory support via nocturnal non-invasive ventilation has been shown to improve survival. However, interventions targeting respiratory muscle performance—such as inspiratory muscle training (IMT)—remain underexplored in DM1 despite being used in other neuromuscular disorders (e.g., Duchenne muscular dystrophy, Pompe disease). Preliminary evidence suggests IMT can be beneficial in neuromuscular conditions with respiratory muscle involvement, especially in slowly progressive conditions. It may enhance ventilation, alleviate symptoms related to hypoventilation, and stabilize lung function [11–17]. However, studies in DM1 are limited, often lacking control groups, adequate sample sizes, or long-term follow-up [18].

This randomized controlled trial aimed to evaluate the feasibility, safety, and efficacy of two IMT modalities—strength and endurance training—in patients with genetically confirmed DM1 over a nine-month intervention period. Based on results of previous studies in various NMDs, we hypothesized that both IMT modalities would lead to significant improvements in maximal inspiratory pressure (MIP) compared to a non-training control group, with endurance training additionally enhancing parameters of ventilatory efficiency such as FVC and MVV.

## Methods

### Study design and setting

This was a prospective, single-center, three-arm randomized controlled trial conducted at the Friedrich–Baur-Institute, Department of Neurology, Ludwig-Maximilians-University Munich, Germany, a primary center for neuromuscular diseases. Ethical approval was obtained (Ref. EK 19-330), and the trial was registered at ClinicalTrials.gov (NCT04052958). All participants provided written informed consent.

### Participants

Eligible participants were adults aged ≥ 18 years with genetically confirmed DM1, capable of giving informed consent and performing home-based inspiratory muscle training. Key exclusion criteria included current use of invasive ventilation, severe untreated sleep apnea, participation in another therapeutic clinical trial, inability to perform pulmonary function tests, inability to provide filled-out questionnaires or patient diaries, or severe mobility limitations.

### Randomization and blinding

Participants were randomly assigned in a 1:1:1 ratio to inspiratory strength training, inspiratory endurance training, or control (no training) using stratified randomization based on age, sex, and disease severity (assessed via MIRS). Due to the nature of the intervention, blinding of participants was not feasible. However, outcome assessors were blinded to group allocation.

### Interventions

Participants in the intervention arms received a Respifit S^®^ inspiratory training device (Biegler GmbH, Austria) for threshold resistance training, the most common and validated method of Inspiratory Muscle Training (IMT) [19]. This commercially available device offers inspiratory muscle strength and endurance training modes with adjustable resistance (0–204 cm H_2_O). It also provides digital feedback on training performance, including downloadable efficacy reports. Patients received instructions during screening and baseline visits, along with ongoing in-person or telephone support for device usage. The strength training was prescribed for 5 days/week in seven 2-min intervals with 1-min rest, starting at 30% of baseline MIP and individually increased by 10–20% based on Borg scale. The endurance training consisted of seven 1-min intervals with 1-min rest, starting at 10% of baseline MVV, increased by 20% every follow-up visit based on perceived exertion. Patients maintained a balloon on the device screen within a defined region by breathing regularly. The adherence to training was monitored using device data and patient diaries. Control participants received usual care without respiratory training. The total study duration comprised a nine-month training program with four interim visits at months 1, 3, 5, and 7 for both intervention groups. For patients allocated to the control group, only a single interim visit at month 5 was conducted to minimize potential training effects, such as those arising from repeated pulmonary function testing. Both groups were instructed to use the device properly and, when necessary, received additional support throughout the study via telemedicine (e.g., telephone or email). For both training groups, the target was set at 35 training intervals per week, totaling 1395 intervals for the entire study duration. Participants completed a study-specific diary, documenting both successful and unsuccessful training intervals, along with their perceived exertion using the Borg scale, which ranges from 0 (no exertion) to 10 (maximum exertion). The goal was defined as a training exertion between 5 and 6. If an exertion of 7 or higher was perceived, resistance was decreased to the previous level at which patients reported an exertion of 5–6. To assess adherence, the number of adequate training sessions recorded by the device was compared with the patient's diary entries. Adherence to inspiratory muscle training was determined by contrasting the prescribed training target with the number of successful training sessions stored on the device. The control group did not perform any inspiratory muscle training.

### Outcomes

The primary outcome was defined as a change in maximal inspiratory pressure (MIP) from baseline to 9 months (EOS). The secondary outcomes were defined as changes from baseline to EOS in spirometry in an upright position [forced vital capacity FVC, including forced expiratory volume in the first second FEV1 and the Tiffeneau-index (rFEV1)], maximal voluntary ventilation (MVV), maximum expiratory pressure (MEP), blood gas analyses, the 6-min-walk-test, and patient-reported outcomes (PROMs), comprising the questionnaires ‘Respicheck’, the ‘Fatigue and Daytime Sleepiness Scale’ FDSS, and ‘Epworth Sleepiness Scale’ (ESS). We utilized these three types of patient-reported outcome measures to capture patient-perceived effects of inspiratory muscle training on fatigue, sleepiness, and subjective respiratory function, thereby providing a broader assessment of intervention impact from the patient’s perspective. The Respicheck questionnaire serves as a screening tool for hypoventilation syndrome and includes nine symptom categories, each containing three yes/no questions. A category is positive if at least one question is answered with “yes.” The total score ranges from 0 to 8, with higher scores indicating greater symptom severity [20]. A score of 5 or more suggests a higher probability of hypoventilation in DM1 patients [21]. The FDSS (Fatigue and Daytime Sleepiness Scale) consists of 12 questions related to symptoms associated with daytime sleepiness and sleep-disordered breathing [22]. The Epworth Sleepiness Scale (ESS) is an 8-question assessment of daytime sleepiness. Patients rate their likelihood of dozing during various daily activities on a scale of 0–3. The total score ranges from 0 to 24. While not directly linked to hypoventilation syndrome, the ESS can reflect its effects on daytime sleepiness, as inadequate nighttime ventilation may cause fragmented sleep and excessive daytime sleepiness. Generally, a score above 8 indicates relevant daytime sleepiness, while a score above 10 is considered pathological. Especially ‘ESS’ and ‘Respicheck’ help clinicians assess respiratory and sleep-related symptoms in DM1 patients, potentially indicating the presence of hypoventilation syndrome and its impact on daily functioning. Moreover, patients were advised to complete training diaries that included inspiratory training resistance and the Borg scale. For safety, adverse events and clinically significant changes in respiratory function parameters were collected. Adherence to the training was assessed by the proportion of completed sessions relative to prescribed sessions. Data were collected at baseline, throughout training at four interim visits (months 1, 3, 5, and 7) in the intervention groups at month 5 in the control group, and at the end-of-study (EOS).

### Sample size calculation

Based on an anticipated medium effect size (*f* = 0.5), a power of 0.80, and an alpha of 0.05, a total sample size of 42 was calculated. To account for potential dropouts, 45 participants were planned.

### Statistical analysis

Descriptive statistics and exploratory data analyses for demographic and clinical variables are listed in tables. All statistical analyses were performed using IBM SPSS Statistics (version 27, IBM Corp., Armonk, NY, USA). Microsoft Excel 2020 was utilized for data collection and graphical representation. Categorical data were summarized as frequencies and percentages, while pulmonary function assessments were expressed as percentages of predicted values, adjusted for age, height, and sex using regression models [23–25]. The distribution of each continuous variable was assessed for normality using the Shapiro–Wilk test and by visual inspection of histograms and Q–Q plots. Variables meeting the assumption of normality were analyzed with parametric tests, while non-normally distributed variables were analyzed with non-parametric equivalents. Changes over time within groups were assessed using paired *t*-tests for normally distributed data or Wilcoxon signed-rank tests for non-normally distributed data. Between-group differences in change scores (BL to EOS) were analyzed using ANOVA for parametric data or Kruskal–Wallis tests for non-parametric data, with Bonferroni-corrected pairwise comparisons applied when significant main effects were observed. Given significant baseline differences in maximal inspiratory pressure (MIP), forced vital capacity (FVC), and forced expiratory volume in one second (FEV1), exploratory analyses were conducted using analysis of covariance (ANCOVA) to adjust for baseline values as covariates. Effect sizes were calculated as Cohen’s *d* for parametric data and correlation coefficients (*r*) for non-parametric data. Statistical significance was set at *p* < 0.05 (two-tailed).

## Results

### Participant flow

Of 74 screened participants between October and December 2019, 26 were enrolled, and 25 completed the study (13 females, 13 males; mean age 45.2 ± 9.1 years). One patient refused to continue study assessments after the baseline visit due to lack of time. A CONSORT flow diagram [26] is provided in supplementary Figure S1. All patients were Caucasian. No participants crossed over between groups or were lost to follow-up for reasons related to safety.

### Baseline data

Demographic and clinical baseline characteristics were generally balanced across groups, although significant differences were noted in baseline MIP, FVC, and FEV1 (Tables 1 and 2).

**Table 1:** Demographics

	All (*n* = 26)	Strength (*n* = 9)	Endurance (*n* = 9)	Control (*n* = 8)	*p* value*
Sex m/f	17/9	6/3	6/3	5/3	
Age, years	42.35 ± 12.3	42.11 ± 10.09	36.67 ± 11.89	49.00 ± 12.92	0.115
Height, cm	173.04 ± 9.29	168.22 ± 9.56	174.78 ± 7.08	176.50 ± 9.94	0.146
Weight, kg	70.08 ± 16.96	71.89 ± 22.03	70.89 ± 18.34	67.13 ± 8.58	0.844
BMI, kg/m^2^	23.23 ± 4.62	24.99 ± 5.84	22.95 ± 4.62	21.56 ± 2.33	0.558
CTG-repeats, number	405.76 ± 273.94	337.78 ± 224.04	380.63 ± 242.2	541.25 ± 369.32	0.471
Disease duration, years	19.85 ± 9.61	18.67 ± 8.02	16.33 ± 8.23	25.13 ± 11.38	0.154
MIRS-score					0.692
1	0	0	0	0	
2	1	0	1	0	
3	1	0	1	0	
4	6	4	0	2	
5	18	5	7	6	
Noninvasive ventilation, *n*	7	2	4	1	

All data were parametric and are presented as mean ± SD. *Between-group comparisons were performed using ANOVA; within-group comparisons used paired *t*-test

**Table 2:** Baseline assessments

	All (*n* = 26)	Strength (*n* = 9)	Endurance (*n* = 9)	Control (*n* = 8)	p*
MIP, %pred	98.37 ± 41.1	103.38 ± 38.1	67.08 ± 19.1	127.95 ± 41.0	0.008
FVC, l	3.38 ± 0.7	3.30 ± 0.5	3.08 ± 0.6	3.82 ± 0.8	0.072
FVC, %pred	81.69 ± 16.0	83.42 ± 8.8	71.12 ± 17.5	91.62 ± 14.7	0.021
FEV1, %pred	80.12 ± 15.9	82.8 ± 9.1	68.02 ± 17.4	90.79 ± 11.5	0.005
rFEV1, %	0.83 ± 0.1	0.84 ± 0.0	0.82 ± 0.1	0.83 ± 0.1	0.558
MVV, l/min	73.01 ± 25.5	79.60 ± 30.3	58.74 ± 9.2	83.832 ± 8.6	0.096
MEP, %pred	55.18 ± 20.6	60.10 ± 14.8	51.62 ± 23.4	53.66 ± 24.4	0.680
pH	7.43 ± 0.0	7.43 ± 0.0	7.43 ± 0.0	7.43 ± 0.0	0.991
pCO_2_, mmHg	38.1 [35.0; 41.9]	37.4 [35.7; 41.25]	40.6 [24.2; 41.8]	38.5 [33.3; 42.8]	0.975
pO_2_, mmHg	74.9 [69.8; 78.2]	77.7 [74.7; 78.5]	70.9 [64.5; 78.9]	72.3 [67.8; 78.2]	0.182
6MWT, %pred	71.41 ± 16.4	79.05 ± 11.64	66.89 ± 19.07	67.91 ± 16.59	0.302
Respicheck, score	4.04 ± 1.5	4.00 ± 1.9	3.75 ± 1.4	4.38 ± 1.3	0.743
FDSS, score	11.54 ± 3.9	10.67 ± 4.1	10.89 ± 3.3	13.25 ± 4.0	0.332
ESS, score	12.54 ± 4.2	12.00 ± 5.0	12.33 ± 3.2	13.38 ± 4.9	0.801

Data are presented as mean ± SD (parametric) or median [IQR 25%; 75%] (non-parametric). *Between-group comparisons were performed using ANOVA for parametric or Kruskal–Wallis for non-parametric test, as appropriate

### Primary outcome

**Fig. 1:**
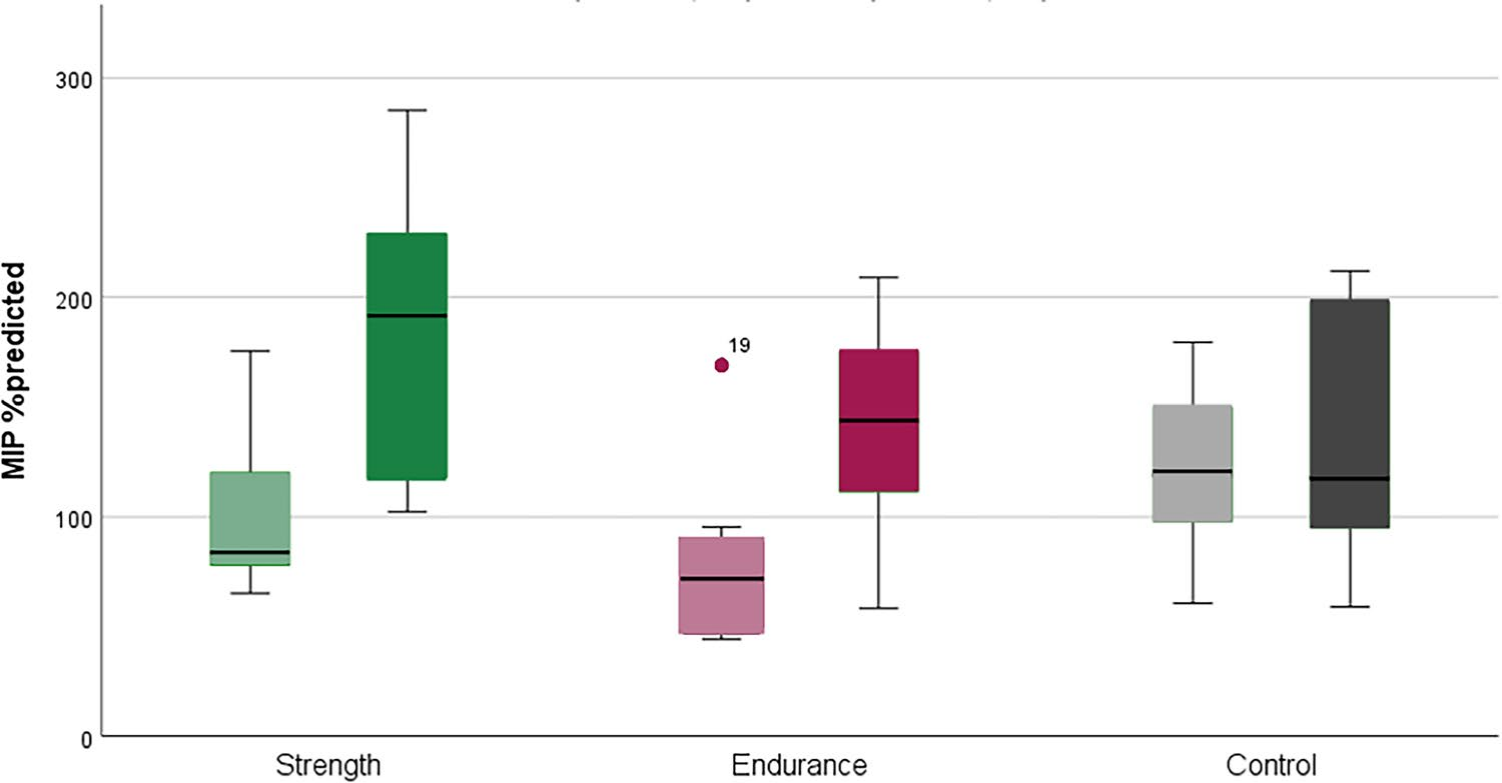
Boxplot change in MIP from BL (Month 0, M0) to EOS (Month 9, M9)

**Fig. 2:**
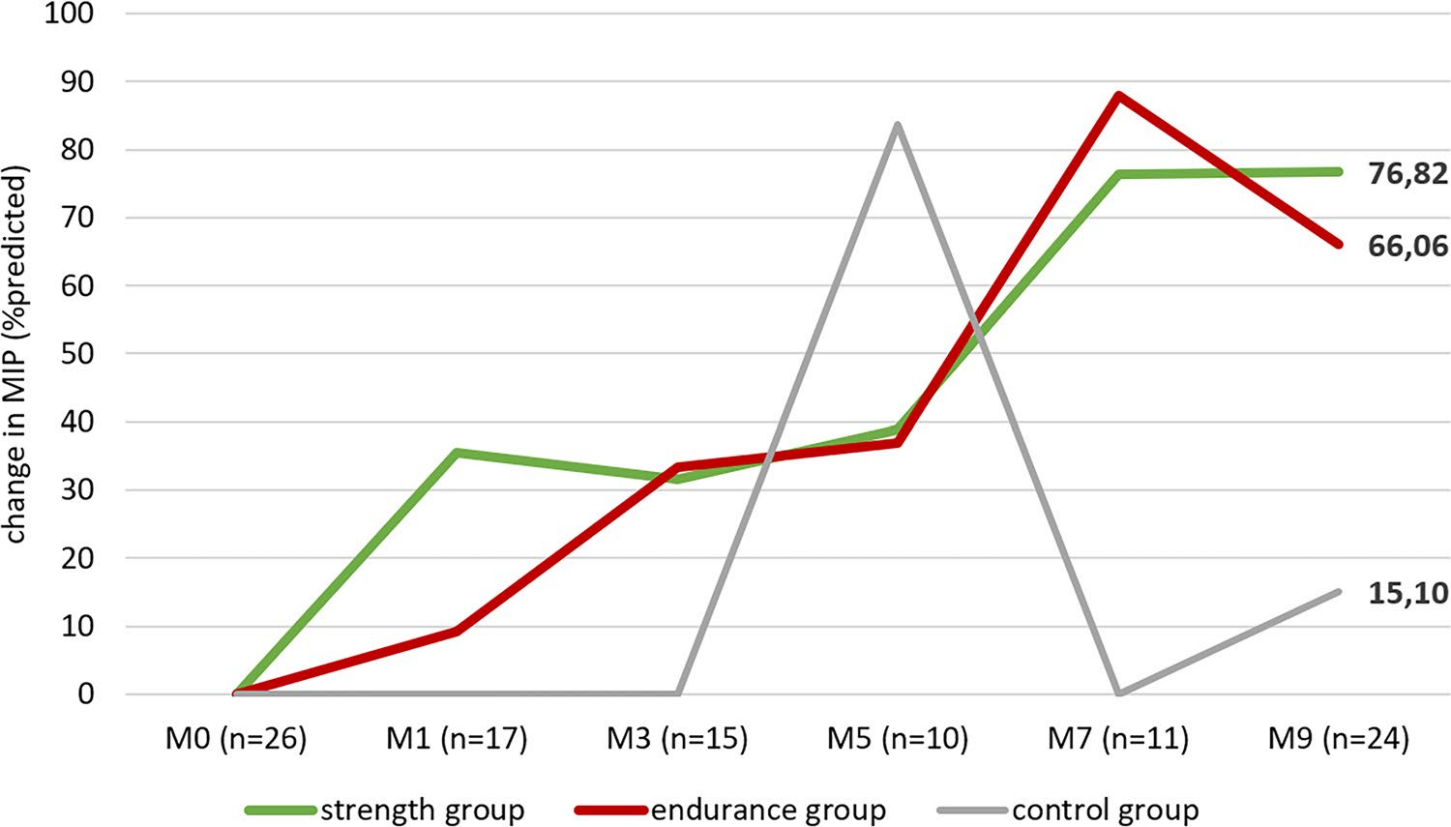
Change in MIP from BL (Month 0, M0) to EOS (Month 9, M9). Note: due to the restrictions during the COVID-19 pandemic, only a total of 10 participants attended at visit month 5, and only one of them was a patient from the control group

Both training groups showed significant improvement in our predefined primary outcome parameter MIP compared to the control group (Table 3). Patients in the strength training group increased their MIP by 76.82% of the target value (*p* = 0.007), the endurance group by 66.06% (*p* < 0.001), both with large effect sizes, and the control group by only 10.49% (n.s.). (Figs. 1 and 2, Table 3, supplements Tables 1a–1c). Pairwise comparisons confirmed significant group differences, both with strong effect sizes (strength-control *r* = 0.677; endurance-control *r* = 0.691). No significant difference was found between the two training groups (*p* = 0.987).

**Table 3:** Outcome—changes from baseline (post–pre), all groups

	Strength (*n* = 8)	Endurance (*n* = 9)	Control (*n* = 8)	p*
MIP, %pred	76.82 ± 57.6	66.06 ± 38.9	10.49 ± 29.0	0.006
FVC, %predicted	1.24 ± 6.7	6.63 ± 8.1	1.74 ± 3.9	0.175
FEV1, %predicted	2.99 ± 4.8	8.04 ± 11.2	0.15 ± 4.8	0.290
rFEV1, %	5.17 ± 14.5	− 0.39 ± 5.8	0.17 ± 3.1	0.797
MVV, %predicted	12.08 ± 15.7	56.18 ± 29.6	5.59 ± 7.0	0.002
MEP, %predicted	6.58 ± 12.3	10.90 ± 14.2	6.13 ± 7.3	0.670
pH	− 0.02 [− 0.32; 0.07]	− 0.08 [− 0.47; 0.00]	− 0.19 [− 0.34; 0.06]	0.830
pCO_2_	1.72 [− 4.07; 3.36]	4.68 [− 5.5; 10.13]	6.31 [4.19; 8.68]	0.489
pO_2_	− 10.39 [− 7.2; − 4.9]	− 1.41 [− 12.8; 6.3]	− 0.11 [− 3.2; 14.5]	0.055
6MWT, %predicted	1.42 ± 6.2	2.92 ± 3.2	− 1.84 ± 3.6	0.150
Respicheck, score	− 1.1 ± 1.5	0.1 ± 1.1	− 0.9 ± 0.7	0.094
FDSS, score	− 0.75 ± 2.6	− 0.67 ± 3.4	− 1.00 ± 1.2	0.968
ESS, score	− 1.6 ± 3.6	− 3.0 ± 2.3	− 2.6 ± 2.5	0.308

Data are presented as mean ± SD (parametric) or median [IQR 25%; 75%] (non-parametric). *Between-group comparisons were performed using ANOVA for parametric or Kruskal–Wallis for non-parametric test, as appropriate

### Secondary outcomes

Among the secondary outcomes, only the endurance group demonstrated a significant increase in FVC, with a mean change of + 6.6% predicted (*p* = 0.039), although between-group differences were not statistically significant. MVV improved markedly in the endurance group (+ 56.2%, *p* < 0.001), with significant differences compared to both the strength (*p* = 0.004) and control groups (*p* = 0.002). Group differences were significant (*p* = 0.002), with pairwise comparisons showing significance between endurance and strength (*p* = 0.004) and endurance and control (*p* = 0.002), both with strong effect sizes (endurance-strength *r* = 0.673; endurance-control *r* = 0.768). No significant within- or between-group changes were observed in MEP. Capillary blood gas parameters changed significantly in pO_2_ in the strength group with a strong effect size (*p* = 0.012, Cohen’s *d* = − 1.311), while pCO_2_ changed significantly in the control group also with a strong effect size (*p* = 0.002, Cohen’s *d* = 1.957). Performance in the 6-min walk test showed a modest but statistically significant improvement in the endurance group (*p* = 0.036, Cohen’s *d* = 0.912), while no relevant changes were observed in the other groups.

Regarding PROMs, ESS scores improved significantly in the endurance (*p* = 0.007, Cohen’s *d* = − 1.323) and control (*p* = 0.035, *d* = − 1.025) groups. Respicheck showed improvement in the control group only (*p* = 0.017, Cohen’s *d* = − 1.242). No significant between-group differences were found for any PROM (table S3).

Adherence, defined as ≥ 75% of prescribed sessions, was achieved by the strength group (mean 78.6%) but not the endurance group (52.7%). Higher adherence correlated with greater improvement in MIP (*p* = 0.031; Cohen’s *d* = 1.27) (supplements table S4 and Figs. S2 and S3).

Regarding safety, no serious adverse events occurred. Mild symptoms (dyspnea, dizziness, and headache) were reported in a few cases but did not result in withdrawal or increased perceived exertion. All outcome measures are provided in Table 3, Supplemental Tables S1–S5, and Supplemental figures S2 and S3).

### Correlation and association analyses

Exploratory analyses were conducted to assess the relationship between baseline values and changes in the primary and secondary outcomes, with a particular focus on maximal inspiratory pressure (MIP), forced vital capacity (FVC), and forced expiratory volume in one second (FEV1), given the significant baseline differences observed between groups. When baseline values were included as covariates in the analysis, the main effects for the primary outcome (change in MIP) remained statistically significant, indicating that the observed training effects were not solely attributable to initial group differences. No significant associations were found between baseline FVC or FEV1 and their respective changes over the study period.

Across the entire cohort, higher adherence to the prescribed training protocol was strongly correlated with greater improvement in MIP (*r* = 0.58, *p* = 0.031). In the endurance group, improvement in MVV was moderately associated with improvement in FVC (*r* = 0.46, *p* = 0.041), suggesting linked physiological adaptations in ventilatory capacity. No other significant correlations between baseline values and change scores were observed for MEP, blood gas parameters, 6-min walk distance, or patient-reported outcomes.

## Discussion

To our knowledge, this is the first randomized controlled trial to evaluate the effects of structured respiratory muscle training in patients with myotonic dystrophy type 1 (DM1). Furthermore, it is the first study to directly compare inspiratory strength training and inspiratory endurance training in this population and among those with neuromuscular diseases involving chronic respiratory muscle impairment. Based on previous studies, regular inspiratory resistance training (strength training) has been shown to enhance diaphragmatic strength, which may delay respiratory decline, especially in patients without ventilatory support dependence. Several published studies on late-onset Pompe disease (LOPD) have reported significant increases in MIP and sustained adherence to short- and long-term IMT [11–13]. Similarly, in Duchenne muscular dystrophy (DMD), long-term IMT has been associated with improvements in inspiratory pressure and the maintenance of pulmonary function parameters, especially when initiated in the early stages of the disease [15, 17].

Our findings demonstrate that both training modalities—strength and endurance—led to significant improvements in the primary outcome (MIP), while the endurance training group additionally showed improvements in ventilatory efficiency parameters such as FVC and MVV. Although no significant difference was observed between the two training types in MIP, the endurance group uniquely benefited in secondary outcomes, suggesting distinct physiological adaptations and clinical advantages. With these results, this study is the first to achieve an improvement in FVC and MVV through respiratory muscle training. This finding indicates a potential selective advantage of endurance training for enhancing ventilatory efficiency. According to our findings, endurance training seems to provide more favorable outcomes than conventional strength training, considering not only maximum inspiratory pressure (MIP) but also forced vital capacity (FVC) and maximum voluntary ventilation (MVV).

Adherence was higher in the strength training group, and a strong correlation was found between adherence and MIP improvement. This highlights the clinical importance of monitoring and supporting long-term engagement in respiratory training. Notably, our intervention was well tolerated, and no serious adverse events occurred, supporting its safety profile.

Baseline imbalances in MIP, FVC, and FEV1 were statistically acknowledged and considered in the interpretation of results. While randomization aimed to ensure group comparability, future studies with larger sample sizes should stratify or adjust more rigorously for these parameters. Clinically notably, no significant correlations were observed between disease duration and changes in MIP, FEV₁, MEP, or 6MWT distance. In contrast, longer disease duration was significantly associated with smaller improvements in forced vital capacity (FVC) and maximal voluntary ventilation (MVV), indicating that patients with more advanced disease may have a reduced capacity for improvement in these parameters. Overall, this suggests that disease severity and duration differently influence the potential for respiratory function improvement. We also observed that higher baseline MIRS scores (= higher disease severity) were associated with greater improvements in maximal expiratory pressure (MEP), a finding that remains unexplained given the lack of similar trends in other outcome measures.

Although PROMs were exploratory and not powered for definitive conclusions, their inclusion aimed to reflect the patient-centered scope of the study and to provide valuable insight into symptom domains not fully captured by objective respiratory parameters. The limited and variable changes in PROMs may reflect either the relatively short duration of follow-up or the multifactorial nature of fatigue and sleep disturbances in DM1 [27]. The modest impact on PROMs may reflect the relatively short follow-up, underlying reasons for fatigue or respiratory symptoms (such as fatigue being a core symptom in DM1), or the limited sensitivity of the tools used. Unlike Pompe disease or DMD, however, DM1 is characterized by more complex multisystem involvement and a broader range of extramuscular symptoms, which may affect the efficacy of respiratory training. Most importantly, sleep-disordered breathing, which occurs frequently in DM1 due to central and/or obstructive sleep apnea, can cause symptoms that cannot effectively be treated by respiratory muscle training.

In addition to the examined outcome parameters, it should be emphasized that patient care was primarily provided through telemedicine (telephone and email) outside the defined visits due to restrictions during the COVID-19 pandemic. The care of the participants was highly effective, particularly in discussing and recording side effects and increases in training resistance. This is an important finding of the study, as it demonstrates that telemedicine can effectively provide support during such training, even in unpredictable circumstances.

Although the overall sample size was modest, this trial was powered based on medium effect sizes and generated robust data on the feasibility and direction of treatment effects. Crucially, the reported effect sizes from this study can now serve to inform sample size calculations for future confirmatory trials.

In summary, our findings underscore the feasibility, safety, and potential benefit of respiratory training in patients with DM1. Both strength and endurance protocols can yield clinically meaningful improvements and therefore should be incorporated into the multidisciplinary care of DM1 patients with early signs of respiratory involvement. Future studies should confirm these results in larger, multicenter cohorts and incorporate polysomnography to distinguish between restrictive and obstructive and central respiratory involvement as well as assess long-term clinical outcomes and quality of life.

## Conclusion

This first randomized controlled trial supports the integration of inspiratory muscle training (IMT) into multidisciplinary care for DM1. Regular inspiratory muscle training is a feasible and safe intervention to improve respiratory strength and ventilatory efficiency in patients with DM1, consistent with results observed in other neuromuscular disorders, such as Pompe disease and Duchenne muscular dystrophy. Both training modalities were effective, with endurance training showing broader benefits. This trial offers foundational evidence for the proactive use of IMT in clinical practice and provides effect size estimates for the design of future trials.

## Supplementary Information

Below is the link to the electronic supplementary material.

Supplementary file1 (DOCX 183 KB)

## Data Availability

The anonymized participant data presented here are available upon request from the correspondent author (stephan.wenninger@med.uni-muenchen.de).
