# Supplementary material for: Efficacy and safety of respiratory strength and endurance training in patients with myotonic dystrophy type 1 (DM1): a randomized controlled trial
Source: J Neurol. 2025 Sep 11;272(9):626. doi: 10.1007/s00415-025-13362-z (PMC12426088; doi:10.1007/s00415-025-13362-z)

**Wenninger, S. et al.:**

**Efficacy and safety of respiratory strength and endurance training in patients with myotonic dystrophy type 1 (DM1): A Randomized Controlled Trial**

**Supplements**

**Tables and Figures**

**Table S1a: Change from baseline to EOS for strength group**

|  | **Baseline**  **(n=8)** | **EOS**  **(n=8)** | **Change** | **p** | **Cohen´s *d*** |
| --- | --- | --- | --- | --- | --- |
| **MIP %pred** | 103.38±38.1 | 182.99±67.08 | 76.82±57.6 | 0.007 | 1.334 |
| **FVC *liters*** | 3.30±0.5 | 3.29+0.63 | 0.18±0.3 | 0.754 | -- |
| **FVC %pred.** | 83.42±8.8 | 84.96±12.39 | 1.24±6.7 | 0.617 | -- |
| **FEV1 %pred.** | 82.8±9.1 | 86.1±9.7 | 2.99±4.8 | 0.121 | -- |
| **rFEV1 *%*** | 0.84±0.0 | 0.91±3.65 | 5.17±14.5 | 0.621 | -- |
| **MVV l/min** | 79.60±30.3 | 81.51±29.07 | 12.08±15.7 | 0.106 | -- |
| **MEP %pred.** | 60.10±14.8 | 67.74±11.81 | 6.58±12.3 | 0.172 | -- |
| **pH** | 7.43 [7.40; 7.44] | 7.41 [7.39; 7.42] | -0.02 [-0.32; 0.07] | 0.132 | -- |
| **pCO₂ *mmHg*** | 37.4 [35.7; 41.25] | 38.75 [36.9; 40.7] | 1.72 [-4.07; 3.36] | 0.182 | -- |
| **pO₂ *mmHg*** | 77.7 [74.7; 78.5] | 69.0 [66.1; 75.4] | -10.39 [-7.2; -4.9] | 0.012 | -1.311 |
| **6MWT *%pred*** | 79.05±11.6 | 82.06±13.08 | 1.42±6.2 | 0.537 | -- |
| **Respicheck *score*** | 4.00±1.9 | 2.75±1.91 | -1.1±1.5 | 0.065 | -- |
| **FDSS *score*** | 10.67±4.1 | 9.75±2.38 | -0.75±2.6 | 0.433 | -- |
| **ESS score** | 12.00±5.0 | 9.50±3.51 | -1.6±3.6 | 0.245 | -- |

Data are presented as mean ± SD (parametric) or median [IQR 25%; 75%] (non-parametric). Between-group comparisons were performed using ANOVA for parametric or Kruskal–Wallis for non-parametric test, as appropriate; within-group comparisons used paired t-test or Wilcoxon signed-rank test. d=cohen´s d for statistically significant results

**Table S1b: Change from baseline to EOS for endurance group**

|  | **Baseline**  **(n=9)** | **EOS**  **(n=9)** | **Change** | **p** | **Cohen´s *d*** |
| --- | --- | --- | --- | --- | --- |
| **MIP %pred.** | 67.08±19.1 | 133.14±46.7 | 66.06±38.9 | <0.001 | 1.698 |
| **FVC *liters*** | 3.08±0.6 | 3.35±0.75 | 0.13±0.3 | 0.049 | 1.533 |
| **FVC %pred.** | 71.12±17.5 | 77.75±21.14 | 6.63±8.1 | 0.039 | 0.823 |
| **FEV1 %pred.** | 68.02±17.4 | 76.07±18.42 | 8.04±11.2 | 0.064 | -- |
| **rFEV1 *%*** | 0.82±0.1 | 1.21±0.3 | -0.39±5.8 | 0.673 | -- |
| **MVV l/min** | 58.74±9.0 | 90.28±14.02 | 56.18±29.6 | <0.001 | 1.897 |
| **MEP %pred.** | 51.62±23.4 | 62.51±27.10 | 10.90±14.2 | 0.066 | -- |
| **pH** | 7.43 [7.40; 7.44] | 7.42 [7.40; 7.44] | -0.08 [-0.47; 0.00] | 0.091 | -- |
| **pCO₂ *mmHg*** | 40.6 [24.2; 41.8] | 40.5 [37.4; 43.5] | 4.68 [-5.5; 10.13] | 0.070 | -- |
| **pO₂ *mmHg*** | 70.9 [64.5; 78.9] | 71.07 [66.9; 77.8] | -1.41 [-12.8; 6.3] | 0.799 | -- |
| **6MWT *%pred*** | 66.89±19.07 | 74.94±12.14 | 2.92±3.2 | 0.036 | 0.912 |
| **Respicheck *score*** | 3.75±1.4 | 4.22±1.92 | 0.1±1.1 | 0.763 | -- |
| **FDSS *score*** | 10.89±3.3 | 10.22±4.44 | -0.67±3.4 | 0.576 | -- |
| **ESS score** | 12.33±3.2 | 10.00±1.93 | -3.0±2.3 | 0.007 | -1.323 |

Data are presented as mean ± SD (parametric) or median [IQR 25%; 75%] (non-parametric). Between-group comparisons were performed using ANOVA for parametric or Kruskal–Wallis for non-parametric test, as appropriate; within-group comparisons used paired t-test or Wilcoxon signed-rank test. d=cohen´s d for statistically significant results

**Table S1c: Change from baseline to EOS for control group**

|  | **Baseline**  **(n=8)** | **EOS**  **(n=8)** | **Change** | **p** | **Cohen´s *d*** |
| --- | --- | --- | --- | --- | --- |
| **MIP %pred.** | 127.95±41.0 | 143.05±60.70 | 10.49±29.0 | 0.375 | -- |
| **FVC *liters*** | 3.82±0.8 | 3.89±0.89 | 0.06±0.3 | 0.517 | -- |
| **FVC %pred.** | 91.62±14.7 | 94.38±17.23 | 1.74±3.9 | 0.283 | -- |
| **FEV1 %pred.** | 90.79±11.5 | 90.58±11.87 | 0.15±4.8 | 0.290 | -- |
| **rFEV1 *%*** | 0.83±0.1 | 0.99±0.21 | 0.17±3.1 | 0.627 | -- |
| **MVV l/min** | 83.83±8.6 | 89.72±35.25 | 5.59±7.0 | 0.130 | -- |
| **MEP %pred.** | 53.66±24.4 | 62.27±25.16 | 6.13±7.3 | 0.067 | -- |
| **pH** | 7.43 [7.40; 7.45] | 7.42 [7.40; 7.46] | -0.19 [-0.34; 0.06] | 0.211 | -- |
| **pCO₂ *mmHg*** | 38.5 [33.3; 42.8] | 38.7 [35.4; 46.0] | 6.31 [4.19; 8.68] | 0.002 | 1.957 |
| **pO₂ *mmHg*** | 72.3 [67.8; 78.2] | 71.0 [67.0; 78.0] | -0.11 [-3.2; 14.5] | 0.488 | -- |
| **6MWT *%pred*** | 67.91±16.59 | 67.95±16.61 | -1.84±3.6 | 0.223 | -- |
| **Respicheck *score*** | 4.38±1.3 | 3.73±1.27 | -0.9±0.7 | 0.017 | -1.242 |
| **FDSS *score*** | 13.25±4.0 | 12.86±3.72 | -1.00±1.2 | 0.062 | -0.866 |
| **ESS score** | 13.38±4.9 | 11.29±4.15 | -2.6±2.5 | 0.035 | -1.025 |

Data are presented as mean ± SD (parametric) or median [IQR 25%; 75%] (non-parametric). Between-group comparisons were performed using ANOVA for parametric or Kruskal–Wallis for non-parametric test, as appropriate; within-group comparisons used paired t-test or Wilcoxon signed-rank test. d=cohen´s d for statistically significant results

**Table S2: Blood gas analysis results: changes from BL-EOS and intergroup comparisons**

|  | **Strength (n=8)** | **Endurance (n=9)** | **Control (n=8)** | **p*** | **p^#^** | **p^x^** |
| --- | --- | --- | --- | --- | --- | --- |
| **pH** | -0.08±0.40 | -0.24±0.72 | -0.16±0.30 | 0.907 | 0.665 | 0.766 |
| **pCO2** | -0.89±6.68 | 5.29±19.06 | 6.12±3.13 | 0.125 | **0.043** | 0.378 |
| **pO2** | -13.16±10.04 | -1.40±16.00 | 2.38±8.54 | **0.018** | **0.007** | 0.484 |
| **Hb** | -0.34±6.70 | 0.07±6.16 | -0.48±6.23 | 0.797 | 0.581 | 0.982 |

All data are presented as mean ± SD (minimum–maximum). unless otherwise indicated. Significance of group differences between all three groups (p*). between the strength and control group (p^#^). and between the endurance and control group (p^x^): Univariate ANOVA (for normally distributed data) or Kruskal-Wallis test (for non-normally distributed data).

**Table S3: Patient reported outcome measures (PROMs) BL-EOS**

|  | Strength  n=8 | Endurance  n=9 | Control  n=8 | p* |
| --- | --- | --- | --- | --- |
| **FDSS *score*** | -0.75±2.6 | -0.67±3.4 | -1.00±1.2 | 0.968 |
| **ESS *score*** | -1.6±3.6 | -3.0±2.3 | -2.6±2.5 | 0.308 |
| **Respicheck *score*** | -1.1±1.5 | 0.1±1.1 | -0.9±0.7 | 0.094 |

Data are presented as mean ± SD (parametric) or median [IQR] (non-parametric). Between-group comparisons were performed using ANOVA for parametric or Kruskal–Wallis for non-parametric test, as appropriate; within-group comparisons used paired t-test or Wilcoxon signed-rank test. ESS – Epworth sleepiness scale: At baseline. 17 patients had pathological scores (strength n = 5. endurance n = 7. control n = 5); at follow-up. this number decreased to 10 (strength n = 2. endurance n = 4. control n = 4). FDSS - Fatigue and Daytime Sleepiness Scale: score decreased in 13 patients (strength n = 3. endurance n = 6. control n = 4). increased in 7 (strength n = 4. endurance n = 3. control n = 0). and remained unchanged in 4 patients. Respicheck: a negative trend was observed in 14 patients (strength n = 6. endurance n = 3. control n = 5). a positive trend in 5 (strength n = 2. endurance n = 3). and stable scores in 4 patients.

**Table S4: Training adherence**

|  | **Total**  **n=17** | **Strength Training n=8** | **Endurance Training n=9** | **p*** |
| --- | --- | --- | --- | --- |
| **Adherence**  **M0-M9 (%)** | 63.04 ± 40.9 (2.1 – 119.9) | 78.60 ± 39.7 (12.6 – 119.9) | 52.66 ± 40.5 (2.1 – 115.0) | 0.242 |
| **Adherence**  **M1 (%)** | 70.03 ± 49.5 (1.7 – 137.9) | 111.16 ± 15.4 (91.7 – 137.9) | 33.46 ± 38.8 (1.7 – 116.9) | 0.001 |
| **Adherence**  **M3 (%)** | 64.80 ± 43.7 (1.3 – 134.9) | 80.51 ± 39.7 (4.7 – 134.9) | 52.59 ± 44.9 (1.3 – 107.6) | 0.215 |
| **Adherence**  **M5 (%)** | 59.30 ± 44.8 (0.0 – 134.5) | 57.23 ± 51.3 (0.0 – 134.5) | 61.13 ± 41.3 (0.7 – 108.3) | 0.864 |
| **Adherence**  **M7 (%)** | 64.88 ± 46.6 (0.0 – 135.6) | 81.34 ± 45.5 (0.0 – 135.6) | 52.09 ± 45.7 (0.3 – 124.6) | 0.224 |
| **Adherence**  **M9 (%)** | 57.57 ± 42.7 (0.0 – 117.9) | 61.66 ± 42.8 (0.0 – 115.9) | 53.94 ± 44.8 (0.6 – 117.9) | 0.722 |

Data are presented as mean ± SD (parametric) or median [IQR] (non-parametric). Unpaired t-test (for normally distributed data) or Mann-Whitney U test (for non-normally distributed data).

**Table S5: Training Adherence and correlation with MIP, Spirometry, MVV and 6MWT**

|  | ‚adherent‘  n=6 | ‚non-adherent‘  n=9 | p* |
| --- | --- | --- | --- |
| **MIP *% predicted*** | 81.30±27.4  (54.4 - 127.6) | 45.93±28.1  (3.6 - 105.5) | **0.031**  **d=1.272** |
| **FVC *l*** | 0.29±0.3  (-0.0 – 0.8) | 0.10±0.3  (-0.2 – 0.6) | 0.366 |
| **FVC *% predicted*** | 9.29±9.8  (-2.9 - 23.4) | 0.97±4.3  (-4.9 - 8.0) | 0.145 |
| **MVV *%*** | 46.57±38.2  (0.0 - 103.0) | 35.04±31.3  (0.0 - 82.4) | 0.659 |
| **6MWT *% predicted*** | 0.47±4.6  (-5.6 - 7.5) | 1.67±3.7  (-5.1 - 4.9) | 0.598 |

Data are presented as mean ± SD (parametric) or median [IQR] (non-parametric). d=cohen´s d for statistically significant results.
*) Univariate ANOVA (for normally distributed data) or Kruskal-Wallis test (for non-normally distributed data).

**Figures**

**Figure S1: Patient flow (according to CONSORT guidelines[1])**


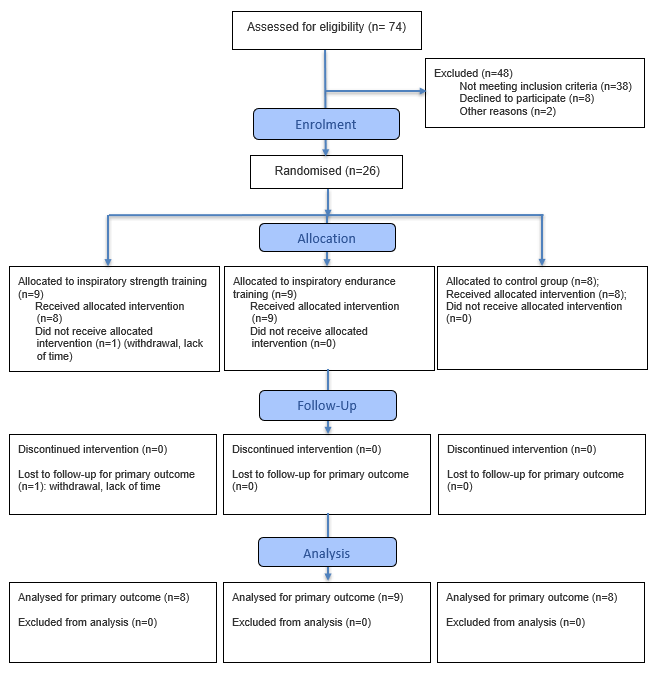


[1] Hopewell S, Chan AW, Collins GS, Hróbjartsson A, Moher D, Schulz KF, et al. CONSORT 2025 Statement: updated guideline for reporting randomised trials. BMJ. 2025; 388:e081123. https://dx.doi.org/10.1136/bmj-2024-081123

**Figure S2: Training adherence for strength and endurance group**

**Figure S3: Training adherence by patient**


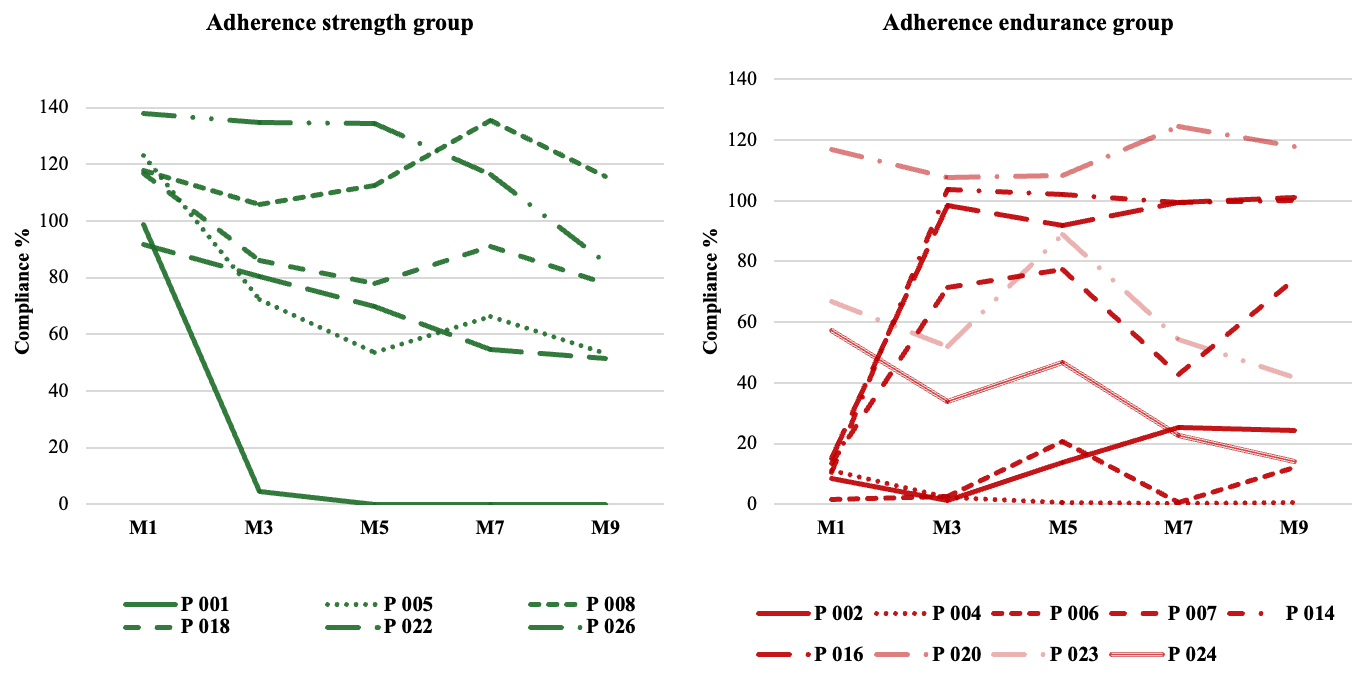

Supplement: Supplementary file 1 — Supplementary file1 (DOCX 183 KB) [file 415_2025_13362_MOESM1_ESM.docx]
